# Two Reliable Methodical Approaches for Non-Invasive *RHD* Genotyping of a Fetus from Maternal Plasma

**DOI:** 10.3390/diagnostics10080564

**Published:** 2020-08-05

**Authors:** Jana Bohmova, Marek Lubusky, Iva Holuskova, Martina Studnickova, Romana Kratochvilova, Eva Krejcirikova, Veronika Durdova, Tereza Kratochvilova, Ladislav Dusek, Martin Prochazka, Radek Vodicka

**Affiliations:** 1 Department of Medical Genetics, University Hospital Olomouc and Faculty of Medicine and Dentistry, Palacky University Olomouc, 775 20 Olomouc, Czech Republic; jana.bohmova@fnol.cz (J.B.); romana.kratochvilova@fnol.cz (R.K.); eva.krejcirikova@fnol.cz (E.K.); martin.prochazka@fnol.cz (M.P.); 2 Department of Obstetrics and Gynecology, University Hospital Olomouc and Faculty of Medicine and Dentistry, Palacky University Olomouc, 775 20 Olomouc, Czech Republic; marek@lubusky.com (M.L.); martina.studnickova@fnol.cz (M.S.); veronika.durdova@fnol.cz (V.D.); tereza.kratochvilova@fnol.cz (T.K.); 3 Department of Blood Transfusion, University Hospital and Palacky University Olomouc, 775 20 Olomouc, Czech Republic; iva.holuskova@fnol.cz; 4 Institute of Biostatistics and Analyses, Masaryk University, 625 00 Brno, Czech Republic; ldusek@med.muni.cz

**Keywords:** non-invasive fetal genotyping, *RHD* gene, cell-free fetal DNA, real-time PCR, QF PCR, Rh blood group system, red blood cell alloimmunization, hemolytic disease of the fetus and newborn

## Abstract

Noninvasive fetal *RHD* genotyping is an important tool for predicting RhD incompatibility between a pregnant woman and a fetus. This study aimed to assess a methodological approach other than the commonly used one for noninvasive fetal *RHD* genotyping on a representative set of RhD-negative pregnant women. The methodology must be accurate, reliable, and broadly available for implementation into routine clinical practice. A total of 337 RhD-negative pregnant women from the Czech Republic region were tested in this study. The fetal *RHD* genotype was assessed using two methods: real-time PCR and endpoint quantitative fluorescent (QF) PCR. We used exon-7-specific primers from the *RHD* gene, along with internal controls. Plasma samples were analyzed and measured in four/two parallel reactions to determine the accuracy of the *RHD* genotyping. The *RHD* genotype was verified using DNA analysis from a newborn buccal swab. Both methods showed an excellent ability to predict the *RHD* genotype. Real-time PCR achieved its greatest accuracy of 98.6% (97.1% sensitivity and 100% specificity (95% CI)) if all four PCRs were positive/negative. The QF PCR method also achieved its greatest accuracy of 99.4% (100% sensitivity and 98.6% specificity (95% CI)) if all the measurements were positive/negative. Both real-time PCR and QF PCR were reliable methods for precisely assessing the fetal *RHD* allele from the plasma of RhD-negative pregnant women.

## 1. Introduction

Alloimmunization of RhD-negative pregnant women by the highly immunogenic fetal D antigen leads to a hemolytic transfusion reaction and hemolytic disease of the fetus and newborn. For this reason, anti-D immune prophylaxis is preventively administered to RhD-negative pregnant women [1,2,3]. By conducting a molecular analysis of cell-free fetal DNA (cffDNA) circulating in the peripheral blood of a pregnant woman, it is possible to determine the fetal *RHD* genotype at an early stage of pregnancy. Early determination of the fetal *RHD* genotype allows for the targeted use of antenatal prophylaxis and the prevention of unnecessary prophylaxis of RhD-negative pregnant women with RhD-negative fetuses. These women are not at risk of immunization and valuable and limited immunoglobulin against D antigen is being spared [4,5].

In the Czech Republic, all pregnant women undergo a red blood cell antibody screening in the first trimester. The screening is positive in about 5% of women (5000 women a year in the Czech Republic), but the clinically significant alloantibody is identified in only about 1.5% of them (1500 women a year). The fetus is at risk for hemolytic disease only if the complementary antigen is present on their erythrocytes. This is the case in about 0.5% of them (500 fetuses a year). The presence of the complementary antigen can be assessed noninvasively by genotyping using cffDNA circulating in the peripheral blood of pregnant women [6].

Identification of the presence of the *RHD* gene in the fetus is based on the detection of unique sequences from the *RHD* gene, especially exon 7 [7]. The *RHD* negative genotype in the White population and thus the absence of D antigen in the erythrocyte membrane is due to the deletion of the *RHD* gene in the homozygous state. Non-cellular fragmented fetal DNA present in the plasma of pregnant women is used for non-invasive prenatal *RHD* genotyping of the fetus. The percentage of cffDNA in the plasma of pregnant women is very variable and most often ranges from 5 to 10% depending on the duration of pregnancy, the size of the placenta, the weight of the woman and fetus, pathological pregnancy, etc. [8]. The aim is to diagnose the fetal *RHD* genotype at an early stage of pregnancy; therefore, it is necessary to use a sensitive method for the detection. The most commonly used method is real-time polymerase chain reaction (PCR) [9,10,11,12,13,14,15]. For non-invasive *RHD* determination of the fetal status, it is also possible to use mini-sequencing [16], emulsion digital PCR (droplet digital PCR, ddPCR) [17,18], quantitative fluorescence PCR (QF PCR) with detection by capillary electrophoresis [19], and massive parallel sequencing [20,21,22].

No representative study has yet been performed in the Czech Republic on a large set of samples to assess the suitability of particular non-invasive *RHD* genotyping methods for introduction into routine clinical practice.

The aim of this study was to assess two methodological approaches for noninvasive fetal *RHD* genotyping on a representative set of RhD-negative pregnant women:

(1) Commonly used real-time PCR (determining fetal *RHD* genotype via TaqMan real-time PCR using internal amplification control).
(2) Endpoint QF PCR (determining fetal *RHD* genotype using endpoint QF PCR using internal amplification control with capillary electrophoresis).

The methodology must be accurate, reliable, and broadly available for implementation into routine clinical practice.

## 2. Materials and Methods

### 2.1. Study Design

This work was a prospective cohort (non-randomized) study. Randomization was not needed because we did not make any conclusions regarding the *RHD* genotype prevalence in the population.

### 2.2. Sample Collection

Pregnant women were serologically tested to be enrolled in the study. The examination for the non-presence/presence of the “D” antigen on erythrocytes of a pregnant woman was performed with two monoclonal diagnostic sera of anti-D class IgM with different clones that do not detect the D category VI (DVI) variant. The examination was performed using the agglutination method on microtiter plates or in a test tube.

Peripheral blood samples of RhD-negative pregnant women, confirmation samples of newborn buccal swabs, and control plasma samples for calibration and optimization were collected in collaboration with the Department of Medical Genetics, the Department of Obstetrics and Gynecology, and the Department of Transfusion Medicine of the University Hospital Olomouc. All of the women enrolled in the study signed an informed consent form approved by the Ethics Committee of the University Hospital Olomouc (approval code: 150/10; approved on 20 September 2010).

The total number of analyzed samples was 337 triplets when it was possible to examine a pregnant woman, a fetus, and a newborn as a control of the fetal *RHD* genotype together. The mothers had already been phenotypically (serologically) and genotypically tested so newborn phenotyping was not performed in this study. The real-time PCR and QF PCR analyses were performed in randomly selected plasma samples taken from RhD-negative pregnant women >18 years old with a singleton pregnancy. The characterizations of the tested RhD-negative pregnant women are in Table 1. Determination of the fetal *RHD* genotype was evaluated in parallel using two methods: TaqMan real-time PCR and endpoint QF PCR.

### 2.3. Sample Preparation and DNA Isolation

All 337 blood samples from RhD-negative pregnant women were collected into two parallel 9 mL tubes (“A” and “B”) containing ethylenediaminetetraacetic acid (EDTA). Anticoagulated blood was placed on ice immediately after collection and was processed within 4 h after sampling. Plasma was separated from the cellular fraction of blood using double centrifugation (2700× *g* for 10 min and 3500× *g* for 20 min). The plasma samples were frozen until further processing at −28 °C. Plasma-cell-free (cf) DNA was isolated in each of the two parallel tubes (“A” and “B”). The DNA isolation of 1 mL of plasma was performed using the QIAamp DNA Mini Kit (Qiagen, Venlo, The Netherlands). The incubation step for the isolation took place at 56 °C, with an elution volume of 65 µL. The isolation of maternal DNA from peripheral blood leukocytes was performed with a Qiacube automated isolator (Qiagen) using the QIAamp DNA mini kit (Qiagen), according to the manufacturer’s instructions. The isolation of the control DNA from newborn buccal swabs was performed using the QIAamp DNA Mini kit (Qiagen), according to the manufacturer’s instructions.

### 2.4. Determination of Fetal RHD Genotype by TaqMan Real-Time PCR Using Internal Amplification Control

Plasma DNA (“A” and “B”) were analyzed from each sample in two parallel reactions (each cffDNA sample was measured four times). The maternal *RHD* genotype was determined from the DNA sample from peripheral blood leukocytes and the fetal genotype was confirmed from a neonatal buccal swab. Specific primers for exon 7 and for the internal control were used to amplify and quantify the multiplex using the TaqMan real-time PCR system, where their sequences were: 5′-GGGTGTTGTAACCGAGTGCTG-3′, forward and 5′-CCGGCTCCGACGGTATC-3′, reverse. The sequence and labeling of the TaqMan probe for *RHD* exon 7 was 5′-FAM-CCCACAGCTCCATCATGGGCTACAA-BHQ1-3′.

The primer and probe sequences from the β-globin gene for the internal total plasma DNA amplification control were GTGCACCTGACTCCTGAGGAGA, forward, CCTTGATACCAACCTGCCCAG, reverse, and 5′-JOE-AAGGTGAACGTGGATGAAGTTGGTGG-BHQ1-3′, TaqMan probe.

The PCR reactions for DNA isolated from the plasma of RhD-negative pregnant women were amplified in a final 25 µL volume. PCR premix contained 12.5 µL of Mastermix (Thermo Scientific Maxima Probe/ROX qPCR Master Mix (2×), Thermo Fisher Scientific, Waltham, MA, USA), 0.15 µL 10× ROX (Thermo Scientific Maxima Probe/ROX qPCR Master Mix (2×)), 1.5 µL forward and reverse multiplexed primers (primer concentration for *RHD* exon 7 was 10–20 pmol/L^−1^, primer concentration for β-globin was 10 pmol/L^−1^) (Sigma-Aldrich, St. Louis, MO, USA), 1 µL of multiplexed TaqMan probes (probe concentration for *RHD* exon 7 was 10–20 pmol/L^−1^ and for β-globin was 10 pmol/L^−1^) (Sigma-Aldrich), and 8.5 µL DNA.

The PCR premix for DNA isolated from the leukocytes of the RhD-negative pregnant women and for control DNA from the neonatal buccal swabs were amplified in a final 12.5 µL volume. The PCR premix contained 6.25 µL of Mastermix (Thermo Scientific Maxima Probe/ROX qPCR Master Mix (2×)), 0.075 µL 10× ROX (Thermo Scientific Maxima Probe/ROX qPCR Master Mix (2×)), 0.75 µL forward and reverse multiplexed primers (primer concentration for *RHD* exon 7 was 10–20 pmol/L^−1^), primer concentration for β-globin was 10 pmol/L^−1^) (Sigma-Aldrich), 0.5 µL of multiplexed TaqMan probes (probe concentration for *RHD* exon 7 was 10–20 pmol/L^−1^ and for β-globin was 10 pmol/L^−1^) (Sigma-Aldrich), 3.25 µL PCR water (Top-Bio, Prague, Czech Republic), and 1 µL DNA.

DNA samples isolated from *RHD*-positive blood and *RHD*-positive fetal DNA isolated from plasma were used as amplification controls. PCR water was used to control the contamination of the PCR premixes. Amplification for all samples was performed in a real-time PCR system Mx3005P (Stratagene, Santa Clara, CA, USA) under the following conditions: 95 °C 15 min, (95 °C 15 s, 60 °C 60 s) 55×. The software Prox-Mx3005P v3.00 Build 311 (Stratagene) was used for the evaluation. The threshold cycle (Ct) values (the number of cycles at which the fluorescence exceeds the threshold value) were determined for each group of samples. Before reading, the measured fluorescence was logarithmized.

Within four parallel measurements, four criteria for evaluating positivity and negativity were set:

- Criterion 1—Positive if all four plasmas were positive, negative if all four plasmas were negative.
- Criterion 2—Positive if three or more plasmas were positive, negative if two or more plasmas were negative.
- Criterion 3—Positive if three or more plasmas are positive, negative if three or more plasmas were negative.
- Criterion 4—Positive if two or more plasmas were positive, negative if three or more plasmas were negative.

### 2.5. Determination of Fetal RHD Genotype with Endpoint QF PCR Using Internal Amplification Control with Capillary Electrophoresis

Plasma DNA (“A” and “B”) was analyzed in two parallel reactions. Specific primers for exon 7 of the *RHD* gene and for *AMELX/Y* sequences were used to amplify and quantify the multiplex using endpoint QF PCR. The sequence and labeling of the primers for *RHD* exon 7 were 5′-HEX-CCCTGGGCTCTGTAAAG-3′, forward, and 5′-CCGGCTCCGACGGTATC-3′, reverse. The primers for the AMELX/Y gonosomal sequences were used as internal amplification controls, where their sequences were: 5′-6FAM-CCCTGGGCTCTGTAAAG-3′, forward, and 5′-ATCAGAGCTTAAACTGGGAAGCT-3′, reverse. The PCR reactions for DNA isolated from the plasma of the RhD-negative pregnant women were amplified in a final 20 µL volume.

The PCR premix contained 10 µL Combi PPP Master Mix (Top-Bio), 1 µL forward and reverse multiplexed primers (primer concentration for *RHD* exon 7 was 10 pmol/L^−1^, primer concentration for *AMELX/Y* was 10 pmol/L^−1^) (Sigma-Aldrich), and 9 μL DNA.

The PCR premix for DNA isolated from leukocytes of the RhD-negative pregnant women and for control DNA from the neonatal buccal swabs were amplified in a final 10 µL volume. The PCR premix contained 5 µL Combi PPP Master Mix (Top-Bio), 0.5 µL forward and reverse multiplexed primers (primer concentrations for *RHD* exon 7 was 10 pmol/L^−1^, the primer concentration for *AMELX/Y* was 10 pmol/L^−1^) (Sigma-Aldrich), 3 µL PCR water (Top-Bio), and 1.5 µL DNA.

DNA samples isolated from the *RHD*-positive blood and *RHD*-positive fetal DNA isolated from plasma were used as the amplification controls. PCR water was used to control for the contamination of PCR premixes. PCR conditions were 95 °C 10 min, (94 °C 30 s, 59 °C 60 s, 72 °C 60 s) 35–40×, 72 °C 10 min, 60 °C 30 min. PCR amplification was performed for all samples in a Thermocycler C 1000 (Bio-Rad, Hercules, CA, USA).

The fluorescence intensity and size of the PCR products were determined using capillary electrophoresis on an ABI PRISM 3130 Genetic Analyzer (Applied Biosystems, Waltham, MA, USA) using polymer POP-4 (Applied Biosystems) and a 47 cm capillary with a diameter of 50 μm. One microliter of PCR product was mixed with 8.5 µL Hi-Di formamide (Applied Biosystems) and the GeneScan 500 TAMRA Size Standard (Applied Biosystems). Each sample of plasma cffDNA was assessed in two capillary electrophoresis conditions. The first injection was for 4 s at 4 kV with electrophoresis for 18 min at 15 kV, and the second injection was for 5 s at 10 kV with electrophoresis for 18 min at 15 kV. The capillary conditions for peripheral blood samples and buccal swab samples were injected for 5 s at 10 kV with electrophoresis for 18 min at 15 kV. The data were analyzed using 310 GeneScan 3.1.2 software (Applied Biosystems). The RFU (relative fluorescence unit) parameter expressed as a peak height was used for quantitative analyses. QF PCR was evaluated with two replicates as we had a limited amount of total plasma DNA.

Within two parallel measurements, two criteria for evaluating positivity and negativity were set:

- Criterion 1—Positive if two plasmas were positive, negative if two plasmas were negative.
- Criterion 2—Positive if one plasma was positive, negative if two plasmas were negative.

### 2.6. Data Collection

Data from plasma DNA were analyzed, evaluated, and collected independently from the newborn DNA ones.

### 2.7. Study Limitation

The data from the evaluation of pregnancy and clinical characteristics associated with plasma or cffDNA concentrations were not available. We did not carry out the molecular analysis of weak *RHD* variants.

### 2.8. Statistical Evaluation

Standard descriptive statistics were applied in the analysis: arithmetic mean with standard deviation (SD) and median with a 5th to 95th percentile range were adopted for the continuous variables and absolute and relative frequencies were adopted for categorical variables.

Fisher’s exact test was applied for the computation of the statistical significance of relations between categorical variables (association analyses).

The predictive power of variables was quantified on the basis of a standard set of statistics: AUC and its statistical significance derived from ROC analysis, specificity, sensitivity, NPV, PPV, and overall accuracy; ROC analysis was applied for the identification of optimal cut-offs of continuous variables as predictors.

Analyses were computed using SPSS 23.0.0.1 (IBM Corporation, 2015, Armonk, NY, USA).

## 3. Results

### 3.1. TaqMan Real-Time PCR

**Figure 1:**
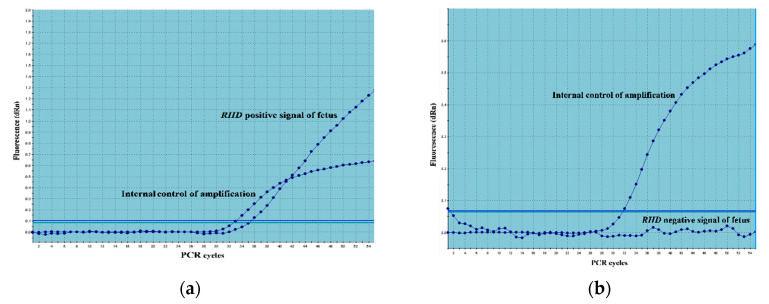
Example of the determination of *RHD*-positive and *RHD*-negative fetus using real-time PCR. Amplification curves show the fluorescence intensity of the probes as a function of the number of PCR cycles (Ct). (**a**) Example of an *RHD*-positive fetus: The *RHD*-positive signal of the fetus was detected by the probe for *RHD* exon 7. The internal control of the amplification was detected using a probe for β-globin. (**b**) Example of an *RHD*-negative fetus: No signal of an *RHD* exon 7 probe was detected. The internal control of the amplification was detected using a probe for β-globin.

Determination of the fetal *RHD* genotype was possible in a total of 333 out of the 337 triplets using real-time PCR. The analysis failed in two plasma samples. The signal intensity of the PCR products from the *RHD* gene and from the internal control β-globin gene was not detectable in these samples. The analysis was not possible, probably due to the low concentration of cffDNA in the plasma, maybe due to DNA degradation. Determination of the fetal *RHD* genotype was not possible in two samples due to repeated *RHD*-positive findings in the maternal DNA. Examples of the output of *RHD*-positive and *RHD*-negative fetuses using the probe for *RHD* exon 7 from real-time PCR are shown in Figure 1.

The predictive power of the diagnostic test and ROC analysis with cut-off Ct values are available in Table 2 and Table 3. It was not possible to evaluate all of the data for any of the four assessed criteria (Table 2 and Table 3). In the real-time PCR method, the greatest power to predict the *RHD* genotype was with criterion 1 (Table 2). ROC analysis and predictive values using the indentified cut-offs showed the best accuracy if the mean of all four parameters with a cut-off (Ct *RHD*-Ct globin) of 16.314 was calculated (Table 3).

### 3.2. End-Point QF PCR

**Figure 2:**
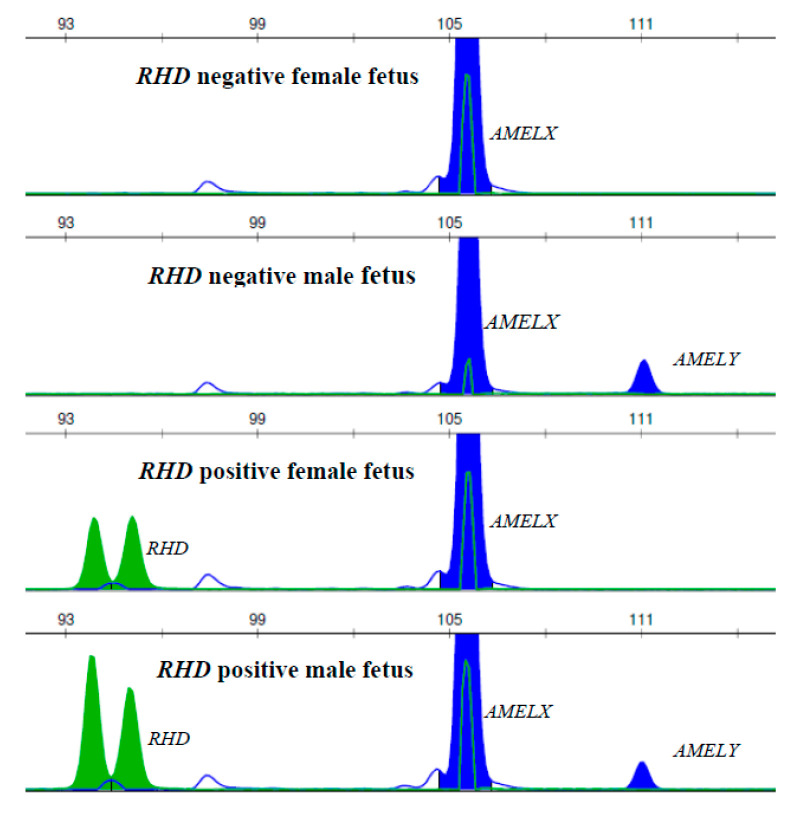
Example of the determination of *RHD*-positive and RhD-negative status and sex of the fetus using endpoint PCR with capillary electrophoresis. The RFU (relative fluorescence unit) parameter is expressed by green peak heights of *RHD* exon 7 and by blue peak heights of *AMELX/Y* gonosomal sequences (internal amplification controls).

Determination of the fetal *RHD* genotype was possible in a total of 335 out of 337 triplets using endpoint QF PCR. Determination of the fetal *RHD* genotype was not possible in two samples due to repeated *RHD*-positive findings in the maternal DNA. Examples of the electrophoretograms of *RHD*-positive and *RHD*-negative fetuses using the probes for *RHD* exon 7 and for *AMELX/Y* are shown in Figure 2.

The predictive power of the diagnostic test and ROC analysis with a cut-off of Ct values are shown in Table 4 and Table 5. It was not possible to evaluate all of the data for any of the two assessed criteria (Table 4 and Table 5). In endpoint QF PCR, the greatest power to predict *RHD* genotype was with criterion 1 (Table 4). The ROC analysis and predictive value using the identified cut-offs showed the best accuracy if the mean of all two parameters with a cut-off (RFU *RHD*/*AMELX*) of 0.023 was calculated (Table 5).

## 4. Discussion

Our study focused on comparing the commonly used TaqMan real-time PCR methodology with the less commonly used endpoint QF PCR with capillary electrophoresis. The sensitivity threshold using the TaqMan real-time PCR system was determined in our previous study. *RHD* calibration was performed using a dilution series of an artificial mixture of *RHD* genotypes. TaqMan real-time PCR was able to capture a 0.22% admixture of an *RHD*-positive heterozygote in an *RHD*-negative homozygote. The sensitivity of QF PCR was assessed using simulations of artificial mosaics of gonosomal sequences in the studies. Even a 0.5% mosaic was reliably captured using this method [23,24]. Both methodologies are therefore suitable for detecting fetal DNA fractions due to the highly sensitive detection limit (below 1%). However, the sensitivity of the methodologies may also be affected by other factors, such as the overall concentration and quality of the fragmented cfDNA. The amount of total cfDNA was also affected by the time lag between the collection of peripheral blood into EDTA tubes and the separation of plasma from plasma cellular components. The amount of total cfDNA increased over time due to the lysis of peripheral leukocytes [25]. The lysis of maternal leukocytes leads to a relative decrease in the fetal fraction and an increase in the maternal fraction, which disrupts the subsequent quantitative analyses.

The typical fetal DNA fraction most often ranges between 5–15% of the total cfDNA across different gestational ages [26,27,28,29,30]. The mean percentage of the fetal fraction could be estimated to 7% (SD = 0.09) in our study using the QF PCR ratio RFU *RHD*/(RFU *AMELX*–RFU *RHD*) from *RHD*-positive samples.

If it is not possible to process the blood within 6 h after the collection, it is advised to use specialized cfDNA preservation tubes (e.g., Cell-Free DNA BCT (Streck, La Vista, NE, USA), CellSave (Cell-Search, Huntingdon Valley, PA, USA), and PAXgene Blood DNA (Qiagen)), which prolong the stability of peripheral leukocytes and allow for storage of whole blood at room temperature for several days [31,32,33].

Barrett et al. reported that the concentration of short cffDNA fragments did not change at room temperature for 72 h when collected in Cell-Free DNA BCT tubes (Streck). In EDTA tubes, the number of long (maternal) DNA fragments increased after 72 h [34]. In our study, blood samples were separated within 4 h after collection, which is consistent with numerous studies that confirm that EDTA collection tubes provide cfDNA stability for at least 6 h, and in some studies up to 24 h [33,35,36,37,38].

Plasma separation conditions can also affect the concentration and quality of cfDNA. The optimized centrifugation method allows for better separation of not only cellular components of blood, but also longer cfDNA fragments of maternal origin from shorter fetal DNA fragments. Commonly used methods are one-step centrifugation for 10 min at 2000–3000× *g* [39,40,41] or two-step centrifugation, where the first step is 1600–3000× *g* for 10 min and the second centrifugation is 10,000–16,000× *g* for 10 min [42,43,44,45,46]. The yield of cffDNA also depends on the chosen methodology for isolating cfDNA. The QIAamp Circulating Nucleic Acid Kit (Qiagen) is currently the most commonly used method for cffDNA isolation.

Jain et al. compared the use of kits for isolating viral DNA and circulating DNA using the QIAamp DSP Virus Kit and the QIAamp Circulating Nucleic Acid Kit. A statistically higher yield of cffDNA was achieved using the QIAamp Circulating Nucleic Acid Kit [47].

In our work, we used the QIAamp DNA Mini Kit (Qiagen, USA), the yield of which was sufficient due to the relatively easy evaluation of the detection of the presence of the *RHD* gene and due to the sensitivity of our methods. Our methodology for *RHD* genotyping using TaqMan real-time PCR and endpoint QF PCR was based on the detection of a unique exon 7 sequence that does not contain the highly homologous *RHCE* gene [7]. Functional (antigenic) variants of the Rh system are caused by insertions/deletions, single nucleotide polymorphisms, or gene conversion between *RHD* and *RHCE* genes [48,49].

In Whites, the RhD-negative genotype is in most cases the result of a homozygous deletion of the entire *RHD* gene [50,51], which was confirmed by our study. The deletion of the *RHD* gene results from uneven recombination between two homologous Rh boxes of paired chromosomes 1, leading to the formation of a hybrid Rh box [52].

It is reported that 12% to 18% of the White population has an RhD-negative genotype [53]. The expression of the D allele in the D/d heterozygote is due to the dominant type of inheritance [51]. In the Black population, *RHD* is 5% negative [53]. There are three common variants in the Black population that do not produce the D antigen. The most common cause of the D negative phenotype (66%) is the presence of an *RHD* pseudogene (*RHDΨ*), which contains a 37 bp nucleotide duplication, resulting in the formation of a premature stop codon in exon 6 [54]. The second variant is the *RHD-CE-D* hybrid gene, which contains nucleotide sequences from the *RHCE* gene, does not produce antigen D, and antigen C is formed abnormally. *RHD-CE-D* hybrid genes probably originated due to the pairing between *RHD* and *RHCE* genes on the same chromosome during meiosis and subsequent gene conversion [55,56]. The third variant is a complete deletion of the *RHD* gene, as in the White population [54].

The detection of the *RHD* gene deletion using exon 7 was shown to be sufficient for the Czech population in this study; for example, in contrast to the Black population, only two (0.6%) discrepancies were found between the mother genotype and the phenotype in our group. We did not test other variants associated with RhD negatives. Our test is diagnostically suitable and usable for such populations in which the vast majority of RhD negativity is caused by the deletion of the entire gene, which is very accurately detectable by exon 7.

For the populations in which several variants are responsible for RhD-negative status, other methods should be considered, for instance, the NGS (Next Generation Sequencing) method [57].

Currently, TaqMan real-time PCR is, due to its high sensitivity and specificity, the most common method for detecting the *RHD* allele of the fetus from free fetal DNA circulating in the peripheral blood of a pregnant woman. A general overview of currently used methods is given in Table 6. In the Czech Republic, real-time PCR [16,17,58] and droplet digital (dd) PCR [16] are used for non-invasive prenatal testing of the fetal *RHD* allele. Our study showed that the endpoint QF PCR method can fully replace real-time PCR. In the case of *RHD*-positive or male-specific fetuses, the amount of fetal fraction can be also relatively easily estimated.

Accuracy characteristics were similar for both methods.

The real-time PCR does not need a capillary electrophoresis separation step and so it takes about 2 h less.

On the other hand, the advantage of QFPCR lies in its direct confirmation of PCR specificity using the length of PCR fragments. In addition, an *AMELY*-specific probe can be used in male fetuses as a cffDNA control and quantificator.

The only currently published studies in the Czech Republic were on small groups of patients. Hromadníkova et al. analyzed a group of 45 pregnant women using real-time PCR [58]. Svobodová et al. compared dd PCR and real-time PCR on a group of 35 pregnant women [17].

Our results are comparable to those published (see Table 6).

## 5. Conclusions

The main goal of this study was to asses and compare possible clinical utilization of two methodological approaches of noninvasive fetal *RHD* genotyping in RhD-negative pregnant women. The study proved there was a minimal discrepancy between the RhD phenotype and the *RHD* genotype for the Czech population and since both the methods showed excellent power to predict the fetal *RHD* genotype from maternal plasma, it is possible to introduce them into clinical practice.

## Figures and Tables

**Table 1:** Characterization of the sample collection.

RhD-Negative Pregnant Women ^1^	*n* = 337	Gestation Week	Gestation Week		Age	Age		BMI	BMI		Ethnic Group of Participants
			Median	Mean		Median	Mean		Median	Mean	
**I. Trimester**	271 (80%)	7–13	12	12.5	18–43	29	30	17–36	23	24.3	Caucasian
**II. Trimester**	66 (20%)	14–23	15	15.5							

^1^ RhD-negative phenotype, *n*—total sum of analyzed samples, BMI—body mass index.

**Table 2:** Predictive power of the diagnostic test combinations.

Criterions	Valid *n*	AUC (*p*-Value)	True Negative	False Negative	False Positive	True Positive	Specificity	Sensitivity	PPV	NPV	Accuracy
Criterion 1	*n* = 217	0.985 (<0.001)	115 (53.0%)	3 (1.4%)	0 (0.0%)	99 (45.6%)	1.000	0.971	1.000	0.975	0.986
Criterion 2	*n* = 322	0.931 (<0.001)	147 (45.7%)	24 (7.5%)	0 (0.0%)	151 (46.9%)	1.000	0.863	1.000	0.860	0.925
Criterion 3	*n* = 303	0.975 (<0.001)	144 (47.5%)	8 (2.6%)	0 (0.0%)	151 (49.8%)	1.000	0.950	1.000	0.947	0.974
Criterion 4	*n* = 327	0.971 (<0.001)	144 (44.0%)	8 (2.4%)	2 (0.6%)	173 (52.9%)	0.986	0.956	0.989	0.947	0.969

PPV—Positive predictive value; NPV—Negative predictive value; AUC—Area under the curve; Criterion 1—Positive if all four plasmas were positive, negative if all four plasmas were negative; Criterion 2—Positive if three or more plasmas were positive, negative if two or more plasmas were negative; Criterion 3—Positive if three or more plasmas were positive, negative if three or more plasmas were negative; Criterion 4—Positive if two or more plasmas were positive, negative if three or more plasmas were negative.

**Table 3:** ROC analysis of diagnostics tests and the predictive power of identified cut-offs.

Ct Differences of RHD7 and Globin	Valid *n*	AUC (*p*-Value)	Cut-Off	True Negative	False Negative	False Positive	True Positive	Specificity	Sensitivity	PPV	NPV	Accuracy
A1-Ct RHD7-Ct globin	*n* = 332	0.898 (<0.001)	−15.821	140 (42.2%)	22 (6.6%)	10 (3.0%)	160 (48.2%)	0.933	0.879	0.941	0.864	0.904
A2-Ct RHD7-Ct globin	*n* = 332	0.902 (<0.001)	−15.378	138 (41.6%)	19 (5.7%)	12 (3.6%)	163 (49.1%)	0.920	0.896	0.931	0.879	0.907
B1-Ct RHD7-Ct globin	*n* = 331	0.869 (<0.001)	−18.073	141 (42.6%)	32 (9.7%)	8 (2.4%)	150 (45.3%)	0.946	0.824	0.949	0.815	0.879
B2-Ct RHD7-Ct globin	*n* = 330	0.848 (<0.001)	−15.984	141 (42.7%)	33 (10.0%)	8 (2.4%)	148 (44.8%)	0.946	0.818	0.949	0.810	0.876
Mean of four parameters	*n* = 330	0.979 (<0.001)	−16.314	144 (43.6%)	8 (2.4%)	5 (1.5%)	173 (52.4%)	0.966	0.956	0.972	0.947	0.961

PPV—Positive predictive value, NPV—Negative predictive value, AUC—Area under the curve. The optimal cut-off was defined by the maximal sum of sensitivity and specificity.

**Table 4:** Predictive power of diagnostic test combinations.

Criterions	Valid *n*	AUC (*p*-Value)	True Negative	False Negative	False Positive	True Positive	Specificity	Sensitivity	PPV	NPV	Accuracy
Criterion 1	*n* = 314	0.993 (<0.001)	140 (44.6%)	0 (0.0%)	2 (0.6%)	172 (54.8%)	0.986	1.000	0.989	1.000	0.994
Criterion 2	*n* = 329	0.976 (<0.001)	140 (42.6%)	0 (0.0%)	7 (2.1%)	182 (55.3%)	0.952	1.000	0.963	1.000	0.979

PPV—Positive predictive value; NPV—Negative predictive value; AUC—Area under the curve; Criterion 1—Positive if two plasmas were positive, negative if two plasmas were negative; Criterion 2—Positive if one or more plasmas were positive, negative if two or more plasmas were negative.

**Table 5:** ROC analysis of diagnostics tests and predictive power of identified cut-offs.

Ratio of RFU RHD/AMELX	Valid *n*	AUC (*p*-Value)	Cut-Off	True Negative	False Negative	False Positive	True Positive	Specificity	Sensitivity	PPV	NPV	Accuracy
RFU RHD/AMELX A1	*n* = 328	0.987 (<0.001)	0.011	145 (44.2%)	2 (0.6%)	2 (0.6%)	179 (54.6%)	0.986	0.989	0.989	0.986	0.988
RFU RHD/AMELX B1	*n* = 327	0.972 (<0.001)	0.001	140 (42.8%)	5 (1.5%)	7 (2.1%)	175 (53.5%)	0.952	0.972	0.962	0.966	0.963
Mean of two parameters	*n* = 326	0.992 (<0.001)	0.023	143 (43.9%)	0 (0.0%)	4 (1.2%)	179 (54.9%)	0.973	1.000	0.978	1.000	0.988

PPV—Positive predictive value, NPV—Negative predictive value, AUC—Area under curve. Optimal cut-off defined by the maximal sum of sensitivity and specificity.

**Table 6:** Methods used for noninvasive prenatal *RHD* genotyping.

Methods	Study	n	Sensitivity in % (95% CI) *	Specificity in % (95% CI) *
Real-time PCR	De Haas 2016 [9]	25,789	99.9 (99.9, 100)	97.7 (97.4, 98.0)
	Haimila 2017 [10]	10,814	100 (99.9, 100)	99.8 (99.6, 99.9)
	Hyland 2017 [11]	599	100 (99.0, 100)	99.6 (97.6, 100)
	Wikman 2012 [14]	3652	97.6 (96.9, 98.2)	98.9 (98.2, 99.4)
	Clausen 2012 [15]	2312	99.9 (99.5, 100)	99.3 (98.7, 100)
	This study	337	97.1	100
Droplet digital PCR	Sillence 2015 [18]	22 ^a^	100	95.5
	Sillence 2015 [18]	24 ^b^	100	100
NGS	Wienzek-Lischka 2015 [21]	4		
	Orzińska 2019 [22]	13		
End-point QF PCR	Kimura 2008 [19]	13		
	This study	337	100	98.6

*n*—number of evaluated participants, a—blood samples from RhD-negative pregnant women collected in EDTA tubes, b—blood samples from RhD-negative pregnant women collected in BCTs tubes, *—when is provided, NGS—Next Generation Sequencing.
